# Supplementary material for: Regulation of the SIRT3/SOD2 Signaling Pathway by a Compound Mixture from Polygonum orientale L. for Myocardial Damage
Source: Pharmaceuticals (Basel). 2024 Sep 27;17(10):1288. doi: 10.3390/ph17101288 (PMC11510516; doi:10.3390/ph17101288)
Supplement: Supplementary file 1 [file pharmaceuticals-17-01288-s001.zip › pharmaceuticals-3138849-supplementary.pdf]

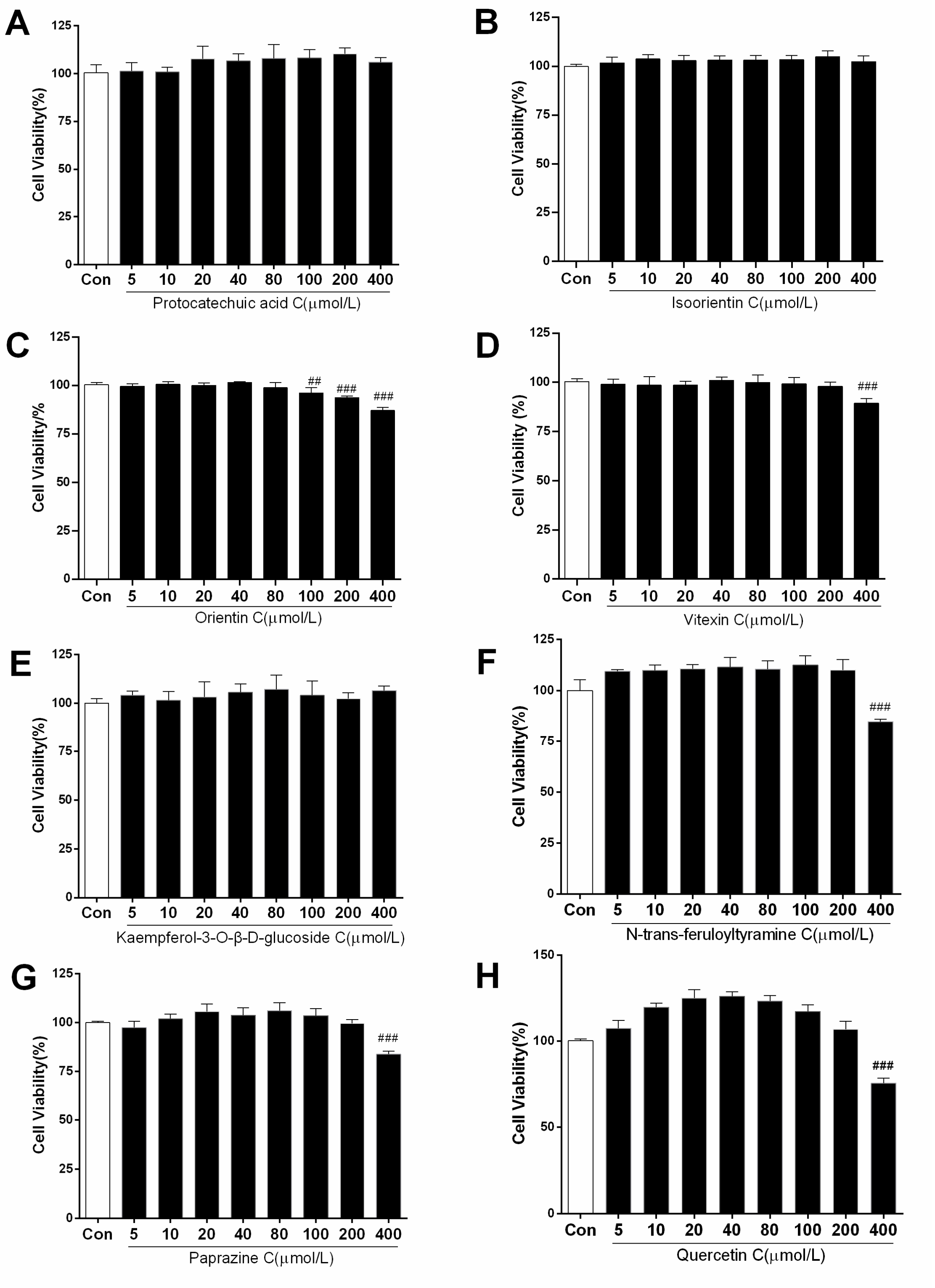

Figure S1. (**A**–**H**) The effect of eight compounds on cell viability in H9c2 cells (H9c2 cells were exposed to the media containing each compound for 24 h, and the cell viability was determined by CCK-8 assay kits; all data were expressed as the mean ± SD, n = 6; # $p < 0.05$, ## $p < 0.01$, ### $p < 0.001$, vs. the Con group).

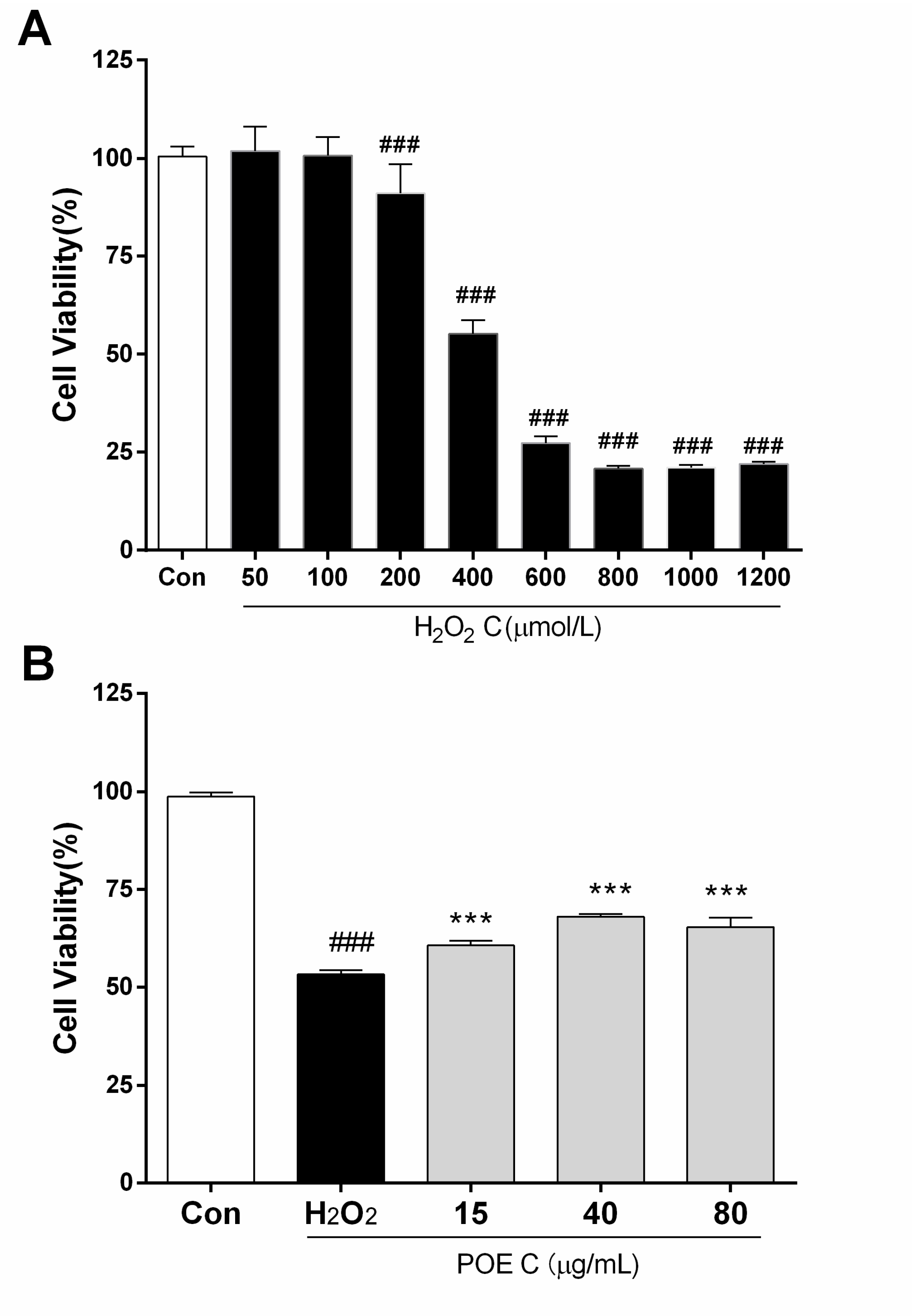

Figure S2. The effect of different concentration of H_2O_2 on H9c2 and the protective effect of POE on H9c2 (**A**: H9c2 cells were exposed to different concentration of H_2O_2 for 0.5 h; **B**: The H9c2 cells were pretreated with POE solution with different concentration for 12 h and then exposed to 400 μM H_2O_2 for 0.5 h; the cell viability was determined by CCK-8 assay kits; all data were expressed as the mean ± SD, n = 6; # $p < 0.05$, ## $p < 0.01$, ### $p < 0.001$, vs. the Con group; *** $p < 0.001$, vs. H_2O_2 group).
